# Perinatal outcomes and congenital anomalies associated with letrozole and natural cycles in single fresh cleaved embryo transfers: A single-center, 10-year cohort study

**DOI:** 10.1016/j.xfre.2022.03.001

**Published:** 2022-03-06

**Authors:** Kazumi Takeshima, Kenji Ezoe, Nami Kawasaki, Hiroko Hayashi, Tomoko Kuroda, Keiichi Kato

**Affiliations:** Kato Ladies Clinic, Tokyo, Japan

**Keywords:** Assisted reproductive technology, congenital anomalies, fresh cleaved embryo transfer, letrozole, perinatal outcomes

## Abstract

**Objective:**

To investigate and compare the safety of letrozole and natural cycles in fresh early embryo transfers.

**Design:**

A retrospective cohort study.

**Setting:**

A large fertility treatment center.

**Patient(s):**

Women who underwent natural and letrozole cycles during fresh early embryo transfer at Kato Ladies Clinic between January 2008 and December 2017.

**Intervention(s):**

None.

**Main Outcome measure(s):**

Perinatal complications and congenital anomalies.

**Result(s):**

No significant differences were observed in pregnancy complications, gestational age, birth weight, small for gestational age, large for gestational age, and congenital anomalies between the the women who underwent natural and letrozole cycles.

**Conclusion(s):**

The perinatal outcomes and congenital anomaly rates associated with letrozole and natural cycles in fresh early embryo transfers were comparable. Therefore, our data support the safe use of letrozole in fresh early embryo transfers in assisted reproductive technology.

**Discuss:** You can discuss this article with its authors and other readers at **https://www.fertstertdialog.com/posts/xfre-d-21-00212**

Letrozole is a type I nonsteroidal aromatase inhibitor that binds competitively to the enzyme aromatase (1). Letrozole suppresses the negative feedback of estradiol to the pituitary gland, after which serum gonadotropin-releasing hormone levels increase and follicular development is induced (2); hence, letrozole is often used in assisted reproductive technology (ART).

Minimal ovarian stimulation with letrozole is considered a patient-friendly strategy because letrozole is a simple and inexpensive oral drug for ovarian stimulation. Recent studies have reported that letrozole-based minimal stimulation is more effective than clomiphene citrate (CC)-based minimal stimulation in patients with polycystic ovarian syndrome (3, 4, 5). Furthermore, few letrozole-related side effects involving the endometrium have been reported (6, 7); thus, letrozole is potentially useful as a regulator of follicular development in the embryo transfer cycle. Nonetheless, a previous study reported that the possibility of fetal motor and congenital cardiac abnormalities could increase through letrozole administration (8); hence, letrozole administration was contraindicated in premenopausal women, and the use of letrozole for follicular development was discontinued worldwide (9). However, that study was not published in a peer-reviewed journal, and several issues regarding the study design, the small cohort size, and the high number of patients who were lost to follow-up have been highlighted. In addition, considering its short half-life of approximately 48 hours, it is theoretically unlikely that the effects of letrozole could extend into the organogenesis period (10). A recent meta-analysis ruled out the possibility of letrozole increasing the number of malformations in children (11). However, most studies have compared the perinatal outcomes, including maternal pregnancy complications, between letrozole and CC cycles; therefore, a comparative study of the natural cycle is warranted. Only 1 study has reported that letrozole cycles do not worsen perinatal or neonatal outcomes, compared with natural cycles in fresh embryo transfers, by analyzing the Japan ART Registry, which contains data from almost all the centers in Japan (12). However, these data lacked details such as a serum hormonal profile, the number of oocytes retrieved, the insemination method, and the quality of the transferred embryo. In addition, there were variations in letrozole use and the culture conditions. In this study, we retrospectively compared the perinatal outcomes and congenital anomalies associated with natural and letrozole cycles in single fresh cleaved embryo transfers (SFCTs) under a uniform protocol at a single institution and investigated the safety of letrozole use in ART.

## Materials and methods

### Study Patients

The clinical records of the women who had undergone natural and letrozole cycles during SFCTs at Kato Ladies Clinic between January 2008 and December 2017 were retrospectively analyzed. The patients’ own oocytes were used during treatment. The patients who underwent preimplantation genetic diagnosis were excluded from the study. This retrospective cohort study was approved by the institutional review board of Kato Ladies Clinic (approval number: 21-14). Written informed consent for the analysis of deidentified data was obtained from all the patients in the study.

### In Vitro Fertilization During Natural and Letrozole Cycles

In the in vitro fertilization (IVF) protocol for a natural cycle, the only pharmacologic intervention was the administration of a gonadotropin-releasing hormone agonist for the induction of final oocyte maturation. Monitoring consisted of an ultrasound scan and a hormone profile; this was usually conducted on the morning of day 10 and/or 12, according to the length of the patient’s cycle. When the leading follicle reached 18 mm in diameter and the estradiol level exceeded 250 pg/mL, ovulation was triggered by the nasal administration of the gonadotropin-releasing hormone agonist buserelin (Suprecur; Mochida Pharmaceutical Co., Ltd., Tokyo, Japan; or BUSERECUR; Fuji Pharma Co., Ltd., Tokyo, Japan). In the IVF protocol for a letrozole cycle, letrozole (Femara, Novartis, Basel, Switzerland; or Letrozole, Fuji Pharma Co., Ltd.) was administered at a dose of 2.5 mg/day on days 3–7, and follicular development was monitored through hormone assay and ultrasonography (13). If follicular development was unsuccessful in this way, oocyte retrieval was canceled, and another protocol of letrozole administration was applied in the subsequent cycle; that is, after checking the hormone levels on the third day of menstruation, letrozole was administered at a dose of 7.5 mg on days 4 and 5, 5 mg on days 6 and 7, and 2.5 mg on days 8 and 9. When the leading follicle reached 18 mm in diameter, ovulation was triggered using buserelin.

Oocyte retrieval was usually performed 30–36 hours after triggering ovulation, using a 21-gauge needle (Kitazato Oocyte Retrieval Needles, Kitazato Corporation, Shizuoka, Japan), generally without anesthesia or follicular flushing. The time interval between the trigger and oocyte retrieval was determined according to the degree of follicular development and hormonal status, particularly the presence of a luteinizing hormone (LH) surge. When an LH surge was not observed at the time of the decision, oocyte retrieval was performed 34–36 hours after the trigger. However, if the LH surge had already occurred at the time of the decision, the timing of oocyte retrieval was determined according to the proceeding degree of the LH surge. For example, if the LH level was 10–20 mIU/mL at the time of the decision, ovulation was triggered immediately, and the eggs were retrieved 30 hours later. Cumulus-oocyte complexes were collected, washed, and subsequently transferred to a human tubal fluid medium (HTF Medium, Kitazato Corporation) with paraffin oil in 5% atmospheric CO_2_ at 37 °C for culturing until either conventional IVF was performed 3 hours later (14) or, in cases of intracytoplasmic sperm injection, denudation was performed 4 hours after oocyte retrieval (15, 16). All embryos were cultured at 37 °C (gas phase: 5% O_2_, 5% CO_2_, and 90% N_2_), with 100% humidity in a water jacket or nonhumidified incubator (APM-30D, Astec Co. Ltd, Fukuoka, Japan). The cleavage-stage embryos were graded using Veeck’s criteria 42 hours after insemination, as previously reported (14).

### Embryo Transfer

At our clinic, the SFCTs were performed on day 2; however, if a patient had a schedule conflict, the SFCTs were performed on day 3. The SFCTs were performed as previously described (17). The cleaved embryos were transferred on days 2 and 3 after oocyte retrieval. The embryo transfer procedure was performed under the guidance of vaginal ultrasound, using a specially designed soft silicone inner catheter (Kitazato ET catheter, Kitazato Corporation). The procedure involved the insertion of a single embryo at a minimal volume in the upper part of the uterine cavity. Dydrogesterone (Duphaston, 30 mg/day; Mylan EPD G.K., Tokyo, Japan) was routinely orally administered during the early luteal phase after transfer in both the groups. Clinical pregnancy was defined according to the ultrasonographic observation of a gestational sac. Information on the maternal and neonatal outcomes was obtained from a questionnaire completed by the patients after their infant’s 1-month examination. At 9 weeks gestation, all the pregnant women were invited to respond to the questionnaire at the second trimester and after delivery. If they did not respond, we contacted them to enquire about their outcomes.

### Study Outcomes

The primary outcomes were pregnancy outcomes, perinatal complications, and major anomalies. Pregnancy outcomes included clinical pregnancy and live birth. Perinatal complications included pregnancy complications (hypertensive disorders of pregnancy; gestational diabetes mellitus; hemolysis, elevated liver enzymes, and low platelet count syndrome; preterm premature rupture of membrane; low-lying placenta; placenta previa; placenta accreta; placenta abruption; and cesarean section) and neonatal outcomes (gestational age [≤27 weeks, 28–31 weeks, 32–36 weeks, 37–41 weeks, and ≥42 weeks], birth weight [<1000 g, 1000–1499 g, 1500–2499 g, and ≥2500 g], small for gestational age, and large for gestational age).

The questionnaire requested information regarding the following: date and mode of delivery, sex, birth weight, and length of the newborn(s); the presence of any birth defect or other anomaly; and pregnancy complications. Live birth was defined as delivery at ≥22 weeks of pregnancy. Preterm delivery was defined as delivery occurring at <37 weeks. Low birth weight and very low birth weight were defined as birth weights <2500 g and <1500 g, respectively. Perinatal mortality was defined as the sum of stillbirths (≥22 pregnancy weeks) and early (within 7 days) neonatal deaths. Small for gestational age and large for gestational age were defined as birth weight below the 10th percentile and above the 90th percentile, respectively, according to Japanese national reference charts for neonates (18). Neonatal outcomes were obtained from the questionnaires completed by the mothers their infant’s 1-month examination. Birth defects were classified using the Q-codes of the International Statistical Classification of Diseases and Related Health Problems, 10th Revision, with classification being performed by reformatting the questionnaire responses (19).

### Statistical Analyses

All statistical analyses were performed using JMP software (SAS Institute, Cary, NC). Proportion data were analyzed using a χ^2^ test. Continuous parameters were compared using a Student’s *t* test. Logistic regression was used to assess the contributing strength of the parameters associated with pregnancy outcomes. Odds ratios and adjusted odds ratios were reported with 95% confidence intervals for each group. Statistical significance was set at a *P* value of <.05.

## Results

### Characteristics of the Study Cohort

**Supplemental Figure 1:**
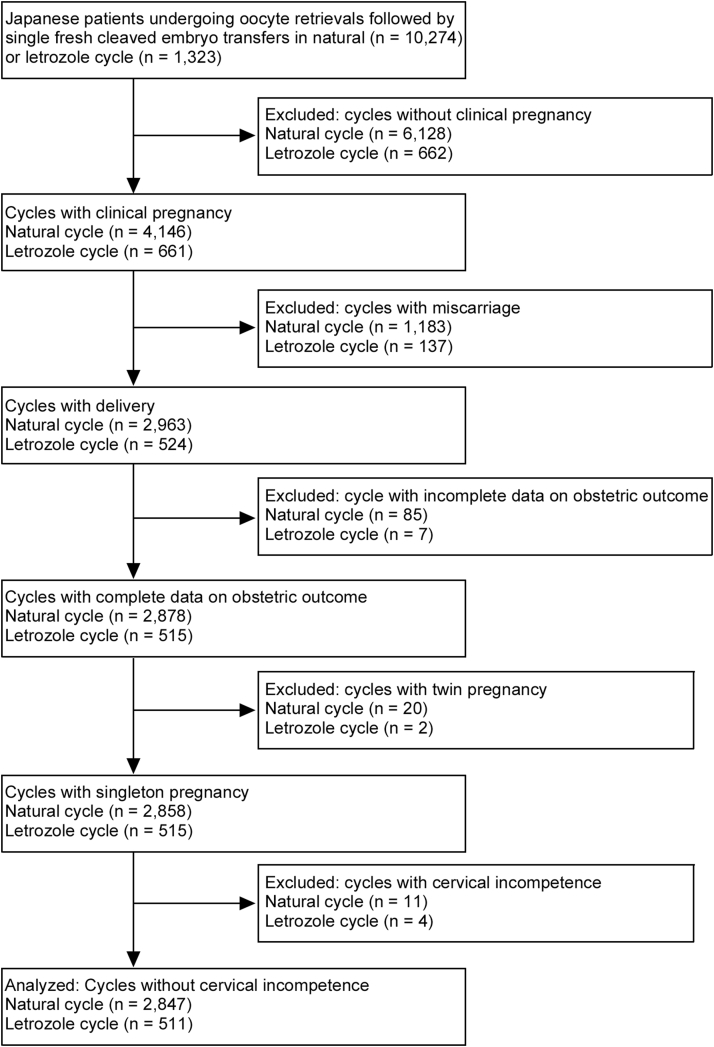
Flowchart describing the method of patient selection, including inclusion and exclusion criteria.

A total of 11,597 SFCTs (natural, 10,274 cycles; letrozole, 1,323 cycles) were performed during the study period (Table 1 and Supplemental Fig. 1 [available online]). The women in the natural group were significantly older than those in the letrozole group (*P*<.0001). The proportion of infertility causes was significantly different between the groups. The serum estradiol level on the day of maturation triggering was significantly lower in the letrozole group than in the natural group (*P*<.0001). However, the number of retrieved oocytes was significantly higher in the letrozole group than in the natural group (*P*<.0001). Embryonic quality also differed between the groups. Although the delivery rate was higher in the letrozole group than in the natural group after univariate analysis (*P*<.0001), no significant difference was observed between the groups after multivariate logistic regression analysis (adjusted odds ratio, 1.060; 95% confidence interval, 0.927–1.213; *P* = .3953) (Supplemental Table 1, available online). We obtained follow-up data on 3,395 cases, which included 3,373 singleton pregnancies (Table 1). Of these, the cases of cervical incompetence were excluded from the analysis; consequently, we analyzed perinatal outcomes and congenital anomalies in 3,358 singleton pregnancies (natural, 2,847 cycles; letrozole, 511 cycles). There was no statistical difference in the stillbirth rate between the natural and letrozole groups (*P* = .5857).

**Table 1:** Cohort characteristics and pregnancy outcomes of the study cohort.

Cycles	Natural	Letrozole	*P* value
Embryo transfer cycles, n	10,274	1,323	
Female age, mean ± SEM	36.2 ± 0.0	33.1 ± 0.1	<.0001
<35, n (%)	4,800 (46.7)	1,049 (79.3)	<.0001
35–39, n (%)	3,294 (32.1)	252 (19.1)	<.0001
≥40, n (%)	2,180 (21.2)	22 (1.7)	<.0001
Body mass index, mean ± SEM	20.4 ± 0.0	20.4 ± 0.1	.8632
Cause of infertility			
Ovulation, n (%)	29 (0.3)	115 (8.7)	<.0001
Tubal factor, n (%)	765 (7.5)	73 (5.5)	.0108
Endometrial factor, n (%)	334 (3.3)	19 (1.4)	.0003
Male factor, n (%)	982 (9.6)	128 (9.7)	.8918
Combined, n (%)	416 (4.1)	73 (5.5)	.0123
Unexplained, n (%)	7,748 (75.4)	915 (69.2)	<.0001
Serum estradiol level (pg/mL), mean ± SEM	295.1 ± 0.9	216.1 ± 2.4	<.0001
No. of oocytes retrieved, mean ± SEM	1.1 ± 0.0	1.3 ± 0.0	<.0001
Insemination method			
Conventional in vitro fertilization	4,241 (41.3)	563 (42.6)	.3752
Intracytoplasmic sperm injection	6,033 (58.7)	760 (57.5)	.3752
No. of blastomeres on day 2, mean ± SEM	5.1 ± 0.0	5.4 ± 0.0	<.0001
Morphological grade on day 2			
Grade 1	614 (6.0)	259 (19.6)	<.0001
Grade 2	4,777 (46.5)	19 (1.4)	<.0001
Grade 3	4,801 (46.7)	1,045 (79.0)	<.0001
Grade 4	82 (0.8)	0 (0)	.0011
Day of the embryo transfer			.1777
Day 2	9,550 (93.0)	1,243 (94.0)	
Day 3	724 (7.0)	80 (6.0)	
Clinical pregnancy, n (%)	4,146 (40.4)	661 (50.0)	<.0001
Deliveries, n (%)	2,963 (28.8)	524 (39.6)	<.0001
Miscarriage, n (%)	1,183 (28.5)	137 (20.7)	<.0001
Follow-up data, n	2,878	517	-
Singleton pregnancies, n	2,858	515	-
Cycles without cervical incompetence, n	2,847	511	-

*Note:* Values are presented as mean ± SEM or n (%).

### Perinatal Outcomes After Natural and Letrozole Cycles in SFCTs

The perinatal outcomes in the live-birth cycles were stratified on the basis of ovarian stimulation (Table 2). Age was significantly higher in the letrozole group than in the natural group (*P*<.0001). In contrast, body mass index and the incidence of pregnancy complications were both comparable between the groups. The cesarean section rate was lower in the letrozole group (*P* = .0006). Gestational age, birth length, birth weight, and infant sex were statistically comparable between the groups. The incidences of infant mortality and birth defects were comparable between the groups.

**Table 2:** Perinatal outcomes in the study cohort, stratified using the ovarian stimulation method.

Cycles	Natural	Letrozole	*P* value
Live birth, n (%)	2,844 (99.9)	510 (99.8)	.5857
Still birth, n (%)	3 (0.1)	1 (0.2)	.5857
**Live birth**			
Female age, mean ± SEM	34.4 ± 0.1	32.6 ± 0.1	<.0001
<35, n (%)	1,781 (62.6)	430 (84.3)	<.0001
35–39, n (%)	903 (31.8)	78 (15.3)	<.0001
≥40, n (%)	160 (5.6)	2 (0.4)	<.0001
Body mass index, mean ± SEM	20.3 ± 0.0	20.3 ± 0.1	.7117
Pregnancy complications, n (%)	214 (7.5)	29 (5.7)	.1403
Hypertensive disorders of pregnancy, n (%)	103 (3.6)	13 (2.6)	.2222
Gestational diabetes mellitus, n (%)	57 (2.0)	10 (2.0)	.9485
HELLP syndrome, n (%)	3 (0.1)	1 (2)	.5852
Preterm premature rupture of membrane, n (%)	6 (0.2)	1 (0.2)	.9459
Low-lying placenta, n (%)	9 (0.3)	1 (0.2)	.6461
Placenta previa, n (%)	28 (1.0)	2 (0.4)	.1907
Placental accrete, n (%)	1 (0.0)	0 (0)	.6719
Placenta abruption, n (%)	5 (0.2)	2 (0.4)	.3242
Other, n (%)	9 (0.3)	0 (0)	.2033
Cesarean section, n (%)	720 (25.3)	93 (18.2)	.0006
Gestational age, weeks, mean ± SEM	39.2 ± 0.0	39.2 ± 0.1	.6845
Gestational age, ≤27 weeks, n (%)	5 (0.2)	2 (0.4)	.3242
Gestational age, 28–31 weeks, n (%)	16 (0.6)	1 (0.2)	.2831
Gestational age, 32–36 weeks, n (%)	127 (4.5)	24 (4.7)	.8095
Gestational age, 37–41 weeks, n (%)	2,687 (94.5)	482 (94.5)	.9781
Gestational age, ≥42 weeks, n (%)	9 (0.3)	1 (0.2)	.6461
Birth length, cm, mean ± SEM	49.1 ± 0.0	49.0 ± 0.1	.9885
Birth weight, g, mean ± SEM	3023.1 ± 7.9	2999.7 ± 17.8	.2408
Birth weight, <1,000 g, n (%)	9 (0.3)	2 (0.4)	.6784
Birth weight, 1,000–1,499 g, n (%)	7 (0.3)	2 (0.4)	.5572
Birth weight, 1,500–2,499 g, n (%)	202 (7.1)	35 (6.9)	.8456
Birth weight, ≥2,500 g, n (%)	2,626 (92.3)	471 (92.4)	.9886
Small for gestational age	135 (4.8)	29 (5.7)	.3650
Large for gestational age	394 (13.9)	60 (11.8)	.2041
Infant sex			
Male, n (%)	1,413 (49.7)	256 (50.2)	.8312
Female, n (%)	1,431 (50.3)	254 (49.8)	-
Infant death, n (%)	3 (0.1)	1 (0.2)	.5852
Birth defect, n (%)	73 (2.6)	18 (3.5)	.2179

*Note:* Values are presented as mean ± SEM or n (%). HELLP= hemolysis, elevated liver enzymes, and low platelet count.

Table 3 shows the multivariate logistic regression analysis of perinatal outcomes after fresh cleaved embryo transfers, including a comparison of the letrozole and natural cycles. The associations of ovarian stimulation with adverse perinatal outcomes adjusted for age, body mass index, cesarean section, and infant sex were assessed using multivariate logistic regression analysis. Both univariate and multivariate logistic analyses revealed that perinatal outcomes were not adversely affected by letrozole-based minimal ovarian stimulation followed by SFCT.

**Table 3:** Multivariate logistic regression analysis for the perinatal outcomes after fresh cleaved embryo transfers in the letrozole cycle, compared with natural cycles.

Outcomes	Univariate analysis		Multivariate analysis	
	Odds ratio (95% confidential intervals)	*P* value	Adjusted odds ratio (95% confidential intervals)^a^	*P* value
Hypertensive disorders of pregnancy	0.696 (0.388–1.249)	.2246	0.896 (0.464–1.732)	.7450
Gestational diabetes mellitus	0.977 (0.496–1.927)	.9485	0.966 (0.459–2.032)	.9280
HELLP syndrome	1.860 (0.193–17.921)	.5911	1.365 (0.112–16.598)	.8070
Preterm premature rupture of membrane	0.929 (0.111–7.734)	.9459	0.869 (0.085–8.832)	.9058
Low-lying placenta	0.619 (0.078–4.895)	.6493	0.510 (0.060–4.325)	.5373
Placenta previa	0.396 (0.094–1.667)	.2066	0.223 (0.038–1.300)	.0952
Placenta abruption	2.235 (0.432–11.553)	.3371	1.442 (0.249–8.369)	.6829
Cesarean section	0.658 (0.517–0.836)	.0006	0.778 (0.595–1.018)	.0670
Preterm delivery (<37 weeks)	1.018 (0.668–1.552)	.9328	1.299 (0.815–2.068)	.2709
Low birth weight (<2500 g)	0.997 (0.699–1.421)	.9886	1.217 (0.818–1.807)	.3323
Small for gestational age	1.190 (0.788–1.797)	.4078	1.373 (0.863–2.185)	.1815
Large for gestational age	0.828 (0.620–1.106)	.2022	0.818 (0.590–1.135)	.2307
Infant death	1.860 (0.193–17.921)	.5911	3.817 (0.244–32.418)	.1680
Birth defect	1.388 (0.821–2.346)	.2199	1.255 (0.706–2.232)	.4383

*Note:* Reference: Natural group.

a Confounders: female age, body mass index, infertility cause, number of blastomeres on day 2, morphological grade on day 2, endometrial thickness on the day of transfer, and infant sex. HELLP= hemolysis, elevated liver enzymes, and low platelet count.

### Detailed Analysis of the Congenital Anomalies

Congenital anomalies were categorized into 11 types (Table 4 and Supplemental Table 2 [available online]). The incidence of each congenital anomaly was similar between the 2 groups. The most frequent congenital anomaly in both the groups was circulatory defects.

**Table 4:** Congenital anomalies in the study cohort, stratified using the ovarian stimulation method.

Cycles	Natural	Letrozole	*P* value
Live birth, n	2,844	510	
Chromosomal abnormalities, n (%)	5 (0.2)	1 (0.2)	.9205
Circulatory, n (%)	31 (1.1)	7 (1.4)	.5788
Nervous, n (%)	1 (0.0)	1 (0.2)	.1704
Digestive systems, n (%)	3 (0.1)	2 (0.4)	.1223
Urogenital, n (%)	8 (0.3)	3 (0.6)	.2642
Musculoskeletal, n (%)	9 (0.3)	4 (0.8)	.1174
Respiratory, n (%)	3 (0.1)	0 (0)	.4631
Reproductive organ, n (%)	8 (0.3)	2 (0.4)	.6724
Other congenital abnormality, n (%)	6 (0.1)	0 (0)	.2992
Unknown malformation, n (%)	3 (0.1)	0 (0)	.4631

*Note:* Values are presented as n (%).

## Discussion

This study found that letrozole does not increase pregnancy complications and congenital anomalies after fresh early embryo transfer, compared with natural cycles in ART. On comparing the natural and letrozole cycles in this study, participants in the letrozole group were observed to be younger. This may be because letrozole is more commonly used in patients with ovulation disorders, whereas natural cycles are occasionally followed in patients with severely depressed ovarian function, in whom ovarian stimulation is ineffective. Regarding pregnancy complications, despite the cesarean section rate being significantly higher in the natural group, no significant difference was observed in the multivariate analysis, suggesting that the difference was not due to letrozole use. There were no significant differences in congenital anomalies. Although the letrozole group exhibited higher pregnancy and delivery rates and lower miscarriage rates than the natural cycle group, the differences were not significant in multivariate analysis; therefore, letrozole cycles did not result in higher live-birth rates than natural cycles (Supplemental Table 2). Because natural and letrozole cycles do not suppress the endogenous LH surge, a system that allows oocyte retrieval 365 days a year is necessary so that oocytes can be retrieved at the appropriate time, even when an endogenous LH surge occurs. Although this work is more demanding for a facility, it poses a lower physical and financial burden on the patient, because ovulation induction is oral and minimal. Furthermore, especially for patients with ovulation disorders, ovarian stimulation, predominantly with follicle-stimulating hormone products, has been the mainstay in ART; however, the risk of ovarian hyperstimulation syndrome has always been a concern. Nevertheless, ovarian stimulation with letrozole does not cause ovarian hyperstimulation syndrome because the average number of eggs retrieved in this study was 1.3, and it does not cause endometrial thinning, unlike CC (6, 20). Thus, letrozole is a highly effective drug for patients with ovulation disorders.

The strength of the present study was in its analysis of a large dataset from a single center. In addition to the large sample size, the use of letrozole, techniques of oocyte retrieval and transfer, and culture conditions were uniform in this study. Therefore, potential bias caused by differences in the detailed conditions that potentially occur in multicenter data collection was not likely.

This study had certain limitations. First, its findings are not comparable to natural pregnancy. However, in a monitoring study of 10% of all deliveries in Japan, the proportion of congenital anomalies remained within a 3% range (21), which is approximately equivalent to that in this study; therefore, it is unlikely that the risk of congenital abnormalities is significantly increased compared with that associated with a natural pregnancy. Second, this study lacked data on the number of previous ART cycles. However, as a general rule, we limited the use of letrozole or natural cycles in fresh cleaved embryo transfer to the first treatment cycle; thus, the patients with repeated ART failures in our clinic were not included in this data. Moreover, this study was limited because of its retrospective design. Furthermore, we conducted the power analysis on the incidence of pregnancy complications and birth defects between the natural and letrozole groups and detected a difference of 97.7% and 90.6%, respectively. However, this study showed powers ranging from 5.4%–87.4% in detecting a difference in each complication between the groups; therefore, the accuracy of the results of some analyses was low because of the small sample size. Therefore, further studies with larger sample sizes are required to validate our findings.

## Conclusion

No significant differences were observed in the perinatal outcomes and the rate of congenital anomalies between letrozole and natural cycles in fresh early embryo transfers. Our findings support the safety of letrozole use in ART. A recent meta-analysis reported that the pregnancy outcome of mild IVF was similar to that of high-stimulation IVF (22), and the demand for low-stimulation cycles is expected to increase in the future because of their lower financial and physical burden on patients. Letrozole is an essential drug for mild IVF cycles, and the collection of further data on ART is anticipated in the future.

## References

[1] Rose B.I., Brown S.E. (2020). A review of the physiology behind letrozole applications in infertility: are current protocols optimal?. J Assist Reprod Genet.

[2] Malloch L., Rhoton-Vlasak A. (2013). An assessment of current clinical attitudes toward letrozole use in reproductive endocrinology practices. Fertil Steril.

[3] Legro R.S., Brzyski R.G., Diamond M.P., Coutifaris C., Schlaff W.D., Casson P. (2014). Letrozole versus clomiphene for infertility in the polycystic ovary syndrome. N Engl J Med.

[4] Balen A.H., Morley L.C., Misso M., Franks S., Legro R.S., Wijeyaratne C.N. (2016). The management of anovulatory infertility in women with polycystic ovary syndrome: an analysis of the evidence to support the development of global WHO guidance. Hum Reprod Update.

[5] Franik S., Eltrop S.M., Kremer J.A., Kiesel L., Farquhar C. (2018). Aromatase inhibitors (letrozole) for subfertile women with polycystic ovary syndrome. Cochrane Database Syst Rev.

[6] Weiss N.S., van Vliet M.N., Limpens J., Hompes P.G.A., Lambalk C.B., Mochtar M.H. (2017). Endometrial thickness in women undergoing IUI with ovarian stimulation. How thick is too thin? A systematic review and meta-analysis. Hum Reprod.

[7] Mitwally M.F., Casper R.F. (2001). Use of an aromatase inhibitor for induction of ovulation in patients with an inadequate response to clomiphene citrate. Fertil Steril.

[8] Biljan M.M., Hemmings R., Brassard N. (2005). The outcome of 150 babies following the treatment with letrozole or letrozole and gonadotropins. Fertil Steril.

[9] Casper R.F., Mitwally M.F. (2012). A historical perspective of aromatase inhibitors for ovulation induction. Fertil Steril.

[10] Tulandi T., DeCherney A.H. (2007). Limiting access to letrozole—is it justified?. Fertil Steril.

[11] Pundir J., Achilli C., Bhide P., Sabatini L., Legro R.S., Rombauts L. (2021). Risk of foetal harm with letrozole use in fertility treatment: a systematic review and meta-analysis. Hum Reprod Update.

[12] Tatsumi T., Jwa S.C., Kuwahara A., Irahara M., Kubota T., Saito H. (2017). No increased risk of major congenital anomalies or adverse pregnancy or neonatal outcomes following letrozole use in assisted reproductive technology. Hum Reprod.

[13] Ezoe K., Miki T., Okimura T., Uchiyama K., Yabuuchi A., Kobayashi T. (2021). Characteristics of the cytoplasmic halo during fertilisation correlate with the live birth rate after fresh cleaved embryo transfer on day 2 in minimal ovarian stimulation cycles: a retrospective observational study. Reprod Biol Endocrinol.

[14] Ezoe K., Ohata K., Morita H., Ueno S., Miki T., Okimura T. (2019). Prolonged blastomere movement induced by the delay of pronuclear fading and first cell division adversely affects pregnancy outcomes after fresh embryo transfer on day 2: a time-lapse study. Reprod Biomed Online.

[15] Ezoe K., Hickman C., Miki T., Okimura T., Uchiyama K., Yabuuchi A. (2020). Cytoplasmic halo characteristics during fertilization and their implications for human preimplantation embryo development and pregnancy outcome. Reprod Biomed Online.

[16] Ohata K., Ezoe K., Miki T., Morita H., Tsuchiya R., Kaneko S. (2019). Blastomere movement post first cell division correlates with embryonic compaction and subsequent blastocyst formation. Reprod Biol Endocrinol.

[17] Nishihara S., Fukuda J., Ezoe K., Endo M., Nakagawa Y., Yamadera R. (2020). Does the endometrial thickness on the day of the trigger affect the pregnancy outcomes after fresh cleaved embryo transfer in the clomiphene citrate-based minimal stimulation cycle?. Reprod Med Biol.

[18] Itabashi K., Fujimura M., Kusuda S., Tamura M., Hayashi T., Takahashi T. (2010). New standard of average size and weight of newborn in Japan. Jap J Pediat.

[19] World Health Organization International statistical classification of diseases and related health problems, 10th Revision, fifth edition, 2016. https://apps.who.int/iris/handle/10665/246208.

[20] Gonen Y., Casper R.F. (1990). Sonographic determination of a possible adverse effect of clomiphene citrate on endometrial growth. Hum Reprod.

[21] Japan Association of Obstetricians and Gynecologists Yokohama City University Congenital Monitoring Center. Results of the survey on congenital defect. https://icbdsr-j.jp/data.html.

[22] Datta A.K., Maheshwari A., Felix N., Campbell S., Nargund G. (2021). Mild versus conventional ovarian stimulation for IVF in poor, normal and hyper-responders: a systematic review and meta-analysis. Hum Reprod Update.

