# Supplementary material for: Perinatal outcomes and congenital anomalies associated with letrozole and natural cycles in single fresh cleaved embryo transfers: A single-center, 10-year cohort study
Source: F S Rep. 2022 Mar 6;3(2):138–44. doi: 10.1016/j.xfre.2022.03.001 (PMC9250119; doi:10.1016/j.xfre.2022.03.001)
Supplement: Supplemental Tables 1 and 2 [file mmc1.docx]

Supplemental Table 1. Multivariate logistic regression analysis for pregnancy outcomes after fresh cleaved embryo transfers in the letrozole cycle

| Outcomes | Univariate analysis | |  | Multivariate analysis | |
| --- | --- | --- | --- | --- | --- |
|  | Odds ratio  (95% confidence intervals) | *P-*value |  | Adjusted odds ratio  (95% confidence intervals)* | *P-*value |
| Clinical pregnancy | 1.618 (1.437–1.821) | <0.0001 |  | 1.015 (0.891–1.155) | 0.8248 |
| Live birth | 1.618 (1.437–1.821) | <0.0001 |  | 1.060 (0.927–1.213) | 0.3953 |
| Miscarriage | 0.655 (0.536–0.800) | <0.0001 |  | 0.889 (0.710–1.114) | 0.3084 |

Reference: Natural group

*Confounder: female age, body mass index, infertility cause, number of blastomeres on day 2, morphological grade on day 2, and endometrial thickness on the day of transfer

Supplemental Table 2. Congenital malformation details

| Malformation type | Diagnosis |
| --- | --- |
| Chromosomal abnormalities | Down syndrome, Sex chromosome anomaly (Triple X syndrome) |
| Circulatory | Ventricular septal defect, atrial septal defect, patent ductus arteriosus, pulmonary atresia with intact ventricular septum, patent foramen ovale, tetralogy of Fallot |
| Nervous | Agenesis of the corpus callosum, microcephaly |
| Digestive systems | Hirschsprung disease, hypertrophic pyloric stenosis, imperforate anus, duodenal atresia |
| Urogenital | Hydronephrosis, multicystic dysplastic kidney, autosomal dominant polycystic kidney |
| Musculoskeletal | Torticollis, arthrogryposis multiplex congenita |
| Reproductive organ | Cryptorchidism, hypospadias |
| Congenital malformation syndrome | CHARGE syndrome, Alagille syndrome, OHVIRA syndrome |
| Other congenital abnormality | Cleft lip/palate |
